# Adolescent With Acute Liver Failure in the Setting of Ethanol, Cocaine, and Ecstasy Ingestion Treated With a Molecular Adsorbent Recirculating System

**DOI:** 10.7759/cureus.9699

**Published:** 2020-08-12

**Authors:** Jacquelin Peck, Nina Replete, Stephanie Melquist, Francisco Flores, Michael Wilsey

**Affiliations:** 1 Anesthesiology, Mount Sinai Medical Center, Miami Beach, USA; 2 Pediatrics, Johns Hopkins All Children's Hospital, St. Petersburg, USA; 3 Internal Medicine, Sanford Medical Center, Fargo, USA; 4 Pediatric Gastroenterology, Cincinnati Children's Hospital Medical Center, Cincinnati, USA; 5 Pediatrics, University of South Florida College of Medicine, Tampa, USA; 6 Pediatric Gastroenterology, Johns Hopkins All Children's Hospital, St. Petersburg, USA

**Keywords:** gastroenterology, pediatric gastroenterology, pediatrics emergency, liver failure, hepatology, transplant hepatology, critical care, liver dialysis

## Abstract

Recreational polypharmacy intoxication is a popular trend, particularly among adolescents and young adults. Acute liver failure is an uncommon complication of drug intoxication and has been described separately among patients intoxicated with ethanol, cocaine, and 3,4-methylenedioxy-methamphetamine (MDMA, ecstasy). Many patients with acute liver failure will die without liver transplant, and management of drug-induced acute liver failure is complicated by the fact that polysubstance abuse may be a contraindication for liver transplant, even among young patients.

Here we report a case of acute liver failure in an adolescent male secondary to recreational intoxication with ethanol, cocaine, and ecstasy. This patient was not a candidate for liver transplantation. We describe successful treatment using a molecular adsorbent recirculating system (MARS®) or “liver dialysis” and review the literature pertaining to management options for this type of patient.

## Introduction

Combination drug use is a well-established and growing trend among adolescents and young adults. Ethanol, cocaine, and 3,4-methylenedioxy-methamphetamine (MDMA, ecstasy) are among the most commonly abused pharmaceuticals both in the United States and worldwide, so understanding potential complications of concomitant use is essential for modern healthcare providers [1]. One study evaluated drug use in electronic dance music festival (EDM)-related hospitalizations and reported ethanol use in 91.5% of patients and MDMA use in 34.0% of patients [2]. In rodent studies, the combination of ethanol and ecstasy induced greater cellular damage and cardiac stress than either substance used alone [3].

Drug-induced acute liver failure (ALF) is a rare but severe complication that has been described following isolated ethanol, cocaine, and MDMA intoxications [4-9]. However, to the authors’ knowledge, no case report currently describes ALF following co-intoxication with these substances. Here we report a case of ALF in an adolescent male secondary to simultaneous recreational use of ethanol, cocaine and MDMA. This patient’s history of polydrug abuse precluded liver transplantation, and treatment therefore included the use of a Molecular Adsorbent Recirculating System (MARS® therapy) or "liver dialysis."

## Case presentation

A 17-year-old male patient was brought by emergency medical services to our institution’s emergency department with altered mental status following witnessed ethanol, cocaine and MDMA consumption at a music concert. The patient had a known history of drug abuse including cocaine, lysergic acid diethylamide, MDMA, marijuana, and ethanol. The patient’s past medical history was otherwise unremarkable, and he had no previous hospitalizations apart from a prior admission to a drug rehabilitation facility. Physical exam at the time of admission was remarkable for tachycardia of 144 bpm (reference range: 60-100 bpm), tachypnea of 40 bpm (reference range: 12-16 bpm), blood pressure of 107/52 mmHg (reference range: 90-120/50-80 mmHg), oxygen saturation of 98% (reference range: 94%-100%), hyperpyrexia of 41.9°C (reference range: 37°C), and a Glasgow coma scale score of 11 (reference range: 15).

**Figure 1:**
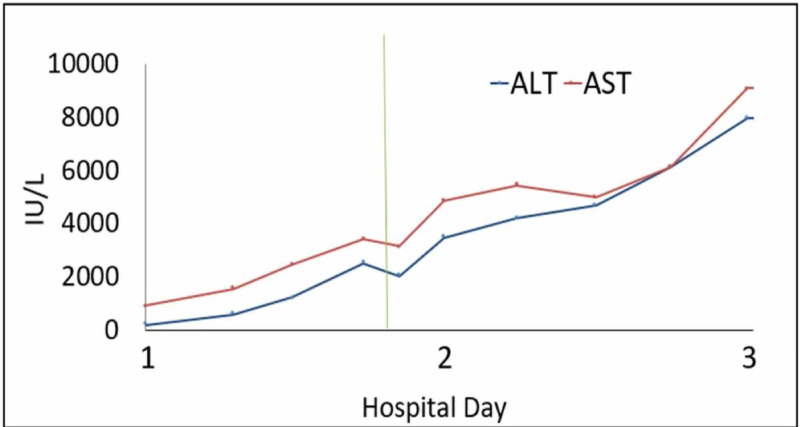
Aspartate transaminase (AST) and alanine transaminase (ALT) progression. The vertical line indicates when MARS therapy was first initiated. MARS, molecular adsorbent recirculating system.

**Figure 2:**
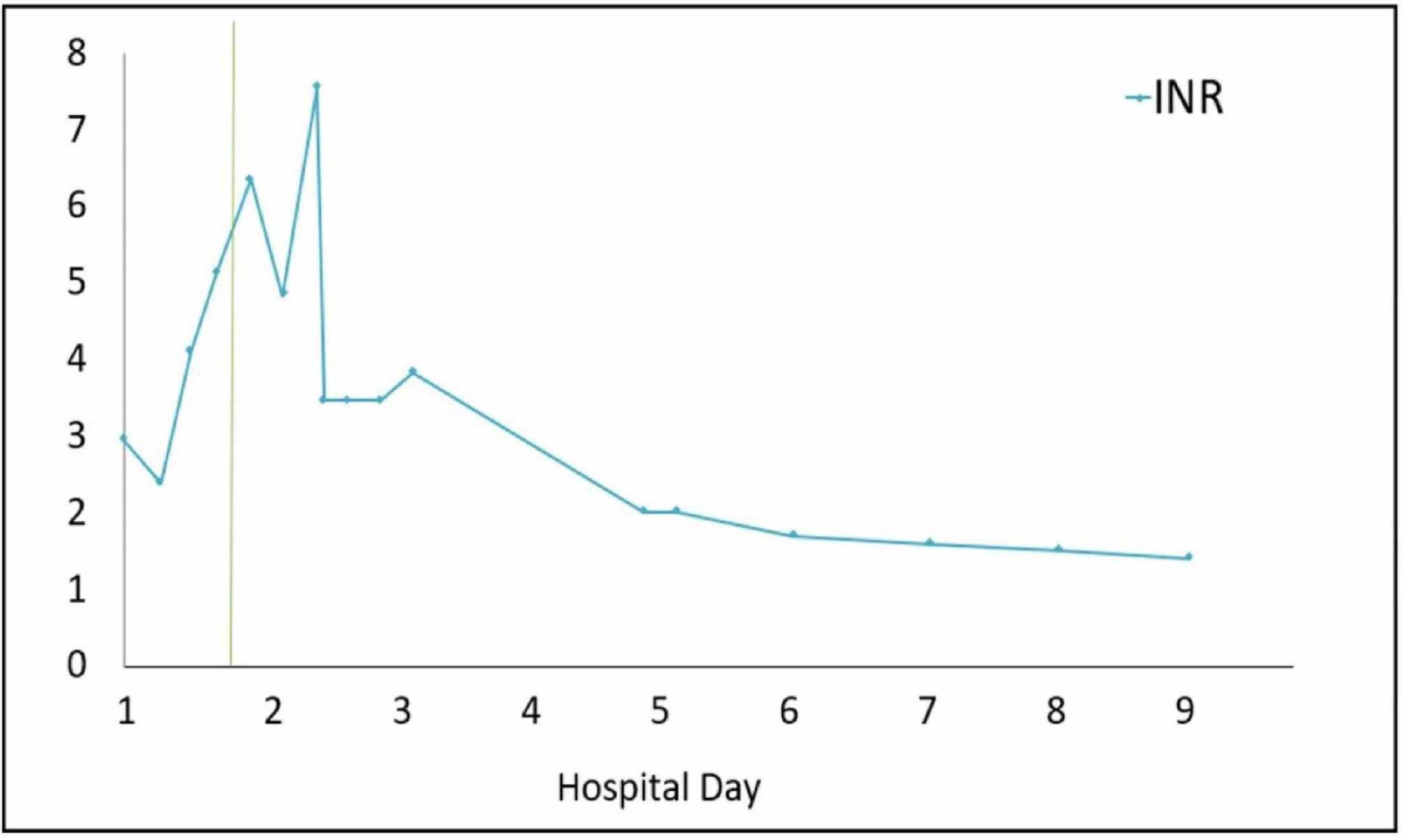
International normalized ratio (INR) progression. The vertical line indicates initiation of first MARS therapy. MARS, molecular adsorbent recirculating system.

**Figure 3:**
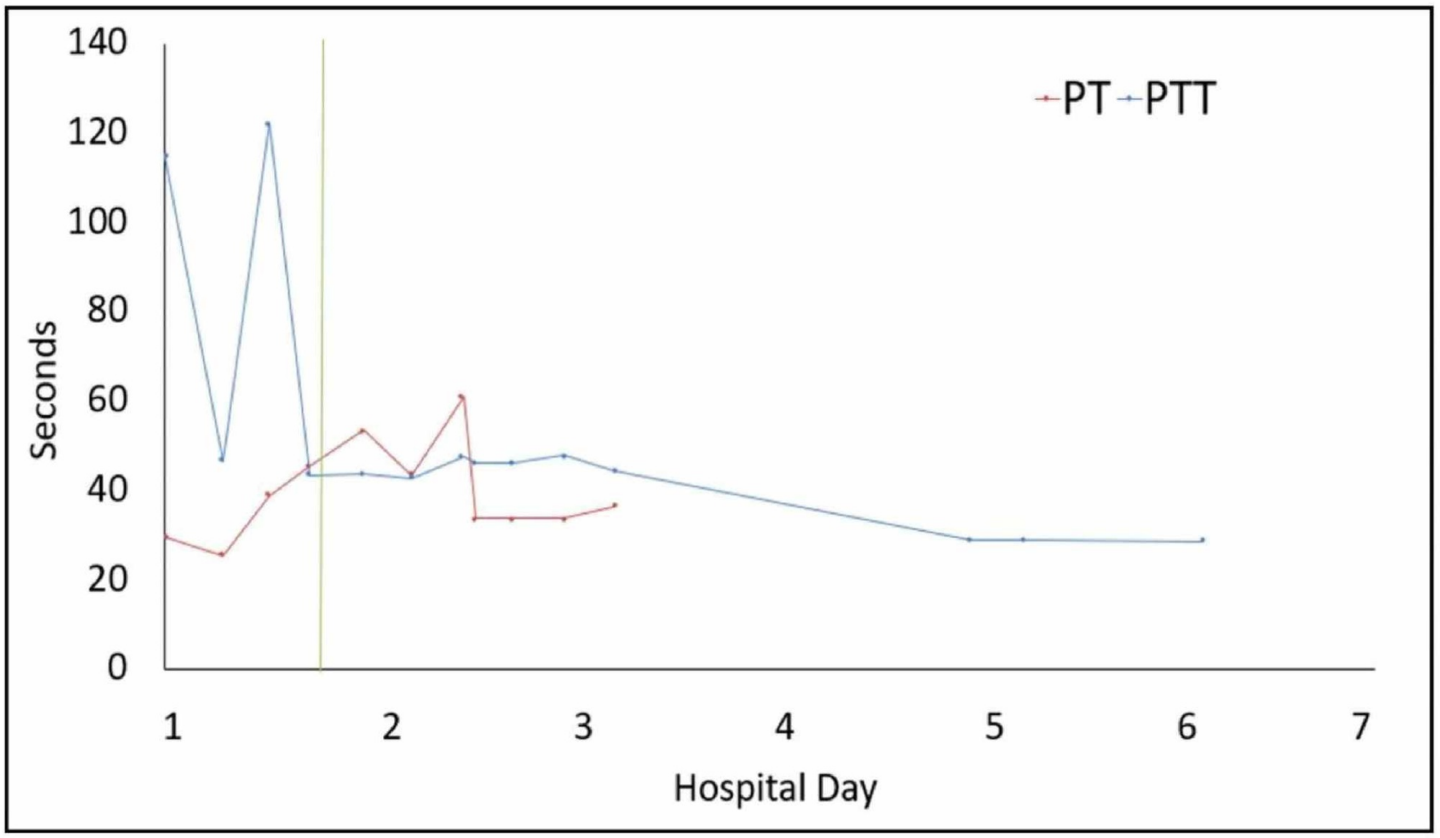
Prothrombin time (PT) and partial thromboplastin time (PTT) progression. The vertical line indicates initiation of first MARS therapy. MARS, molecular adsorbent recirculating system.

Given the patients obtunded state upon presentation, he was admitted to the pediatric intensive care unit. An initial non-contrast CT of his head was negative for any abnormalities. His initial laboratory studies, including a complete blood count (CBC), basic metabolic panel (BMP), and liver panel, revealed numerous derangements including hypoglycemia (glucose 49 mg/dL [reference range: 70-140 mg/dL]], hyperkalemia (10.5 mEq/L [reference range: 3.4-4.7 mEq/L]), and acute kidney injury (AKI) (blood urea nitrogen [BUN] 24 mg/dL [reference range: 5-18 mg/dL], creatinine 2.42 mg/dL [reference range: 0.5-1.0 mg/dL]). He received immediate renal replacement therapy, which did not significantly improve his clinical picture. Repeat labs six hours after admission revealed a rise in alanine aminotransferase (ALT 2,617 U/L [reference range: <31 U/L]) and aspartate aminotransferase (AST 3,352 U/L [reference range: 5-40 U/L]) as well as prolonged prothrombin time PT (>50 seconds [reference range: 8.7-11.5 seconds]), partial thromboplastin time (PTT >120 seconds [reference range: 25-35 seconds]), and international normalized ratio (INR >6 [reference range: 0.8-1.2]). Liver enzymes and were repeated every six hours and trended throughout the remainder of the patient’s hospitalization at our institution (Figures 1-3).

With evidence of acute liver injury, altered mental status, and severely deranged hepatic synthetic function, a diagnosis of ALF was suspected and a facility with liver transplant capabilities was contacted to arrange for transfer after stabilization. On hospital day 1, the patient received one unit each of cryoprecipitate, fresh-frozen plasma (FFP), and platelets, and artificial liver support in the form of MARS therapy was initiated. Following MARS treatment on hospital day 1, the patient's AST and ALT lowered to 2,951 U/L and 2,098 U/L, respectively. The patient was transfused an additional unit of FFP and platelets for correction of his coagulopathy and underwent a second partial cycle of MARS at our institution on day 2. Though AST and ALT levels continued to trend upward following cycles of MARS therapy, the second round of treatment (hospital day 2) resulted in a decrease in AST levels from 5,180 to 4,875 U/L. The patient was transferred to a liver transplant center on day 3 due to unknown trajectory of the hepatic failure. At that facility, he was determined not to be a candidate for liver transplantation because of his history of polysubstance abuse and he continued to receive MARS therapy and renal dialysis as needed.

Over the next seven days at the outside hospital, the patient’s hepatic function spontaneously recovered. His PT, PTT, and INR levels normalized, hepatic support was discontinued, and he recovered baseline mental status. He was discharged on day 10 receiving intermittent renal hemodialysis for the management of his AKI. Hemodialysis was discontinued as an outpatient after he had full recovery of his renal function.

## Discussion

ALF or fulminant hepatic failure is an uncommon condition characterized by coagulopathy, a rapid rise in liver enzymes and INR, and altered mental status (hepatic encephalopathy) in a patient with a previously healthy liver [10]. Triggering causes may include viral infections (hepatitis), autoimmune and vascular disorders, or ingestion of a toxin, most commonly acetaminophen. Due to the rarity of pediatric ALF, current diagnostic criteria, and treatment algorithms are lacking [11]. However, common signs and symptoms may include encephalopathy, hypotension, tachycardia, right upper quadrant tenderness, jaundice, change in liver span, ascites, renal failure, and increased intracranial pressure. Our patient initially presented with tachycardia, fever, and tachypnea, though additional elements of the physical exam may have been clouded by his encephalopathic state. As with our patient, diagnosis is supported by elevated PT/INR, AST, and ALT. Bilirubin, ammonia, and lactate, may also be elevated [10]. Like our patient, many patients also experience moderate or severe hypoglycemia. Approximately 20% of cases are caused by drug-related hepatic injury, but in cases of unknown etiology, viral serologies and autoimmune markers, such as antinuclear antibody or anti-smooth muscle antibody, may be informative [11].

Early care in an intensive care unit and transport to a specialty center is essential for the management of multi-organ dysfunction. Initial medical management consists of crystalloid fluid resuscitation, vasopressor use to maintain a mean arterial pressure above 75 mmHg in adult patients, correction of electrolyte imbalance, and administration of etiology-specific antitoxins when appropriate [12]. In this case, the administration of FFP, cryoprecipitate, and platelets contributed at least partially to the improvements noted in coagulopathy. For this reason, many practitioners advise against the use of these products if coagulopathy is used as a marker for disease status. Liver transplantation is the ultimate treatment and carries a two-year survival rate approaching a 90% following successful transplantation, though this may not be an option for all patients [10]. 

Hepatic support devices may be used as a bridge to transplantation or to provide a short-term physiologic support creating an opportunity for spontaneous recovery of hepatic function. The MARS device consists of two recirculating circuits: one contains an albumin solution with a high affinity for albumin-bound toxins and is separated from the patient by a semipermeable barrier, and the second consists of a hemofiltration system to cleanse the albumin solution [13].

Because of the relative rarity of ALF and the clinical heterogeneity of included patients (with varying etiologies), existing studies are limited and have mixed results. Currently, only one randomized, controlled trial evaluates the use of MARS in ALF [14]. This multi-center trial of 102 patients compares patients receiving standard medical therapy, including liver transplantation, either with or without the addition of MARS therapy. Researchers found no difference in six-month transplant-free survival, overall six-month survival, or overall one-year survival. However, 64.7% of enrolled participants underwent liver transplant (75% of whom underwent surgery within 24-hours), and 14 of the 53 patients (26.4%) of patients randomized to undergo MARS therapy never received the treatment phase because liver transplantation was immediately available. High transplantation rates and short time duration between enrollment and transplantation make it difficult to conclusively evaluate the results or effectively differentiate treatment branches.

Among non-randomized trials, Kantola et al. compared 113 prospectively collected patients receiving MARS therapy with 46 retrospective control patients who did not receive MARS therapy [15]. Survival rates at 28 days and six months were similar in the two groups, though a greater proportion of control patients underwent liver transplant than MARS patients (100% vs 36%). Interestingly, native liver recovery rates were significantly higher in the MARS treatment group than those in the control group (66% vs 40%, p<0.05). However, the etiology of underlying ALF differed between the two groups with a higher proportion of toxin-induced ALF within the MARS group, making a direct comparison difficult.

Based on limited available trials, MARS hepatic support devices have not shown mortality benefit, particularly when compared to liver transplantation. However, given early evidence of improved rates of native liver regeneration, MARS therapy may benefit patients such as ours who have a contraindication for liver transplantation or do not have an available allograft. Both existing studies evaluate the use of MARS therapy among adults with ALF. Further evaluation of MARS therapy in pediatric and adolescent populations is needed.

## Conclusions

Our adolescent patient with polydrug-induced ALF made a full recovery. Early ICU admission and initiation of MARS therapy may have contributed to his positive outcome. As polydrug abuse continues to increase, further research is needed to evaluate the potential role of MARS therapy in the management of this population.
